# BreedingEIS: An Efficient Evaluation Information System for Crop Breeding

**DOI:** 10.34133/plantphenomics.0029

**Published:** 2023-03-14

**Authors:** Kaijie Qi, Xiao Wu, Chao Gu, Zhihua Xie, Shutian Tao, Shaoling Zhang

**Affiliations:** Jiangsu Engineering Research Center for Pear, National Key Laboratory of Crop Genetics & Germplasm Enhancement and Utilization, Nanjing Agricultural University, Nanjing 210095, China.

## Abstract

Crop breeding programs generate large datasets. Thus, it is difficult to ensure the accuracy and integrity of all the collected data in the breeding process. To improve breeding efficiency, we established an open source and free breeding evaluation information system (BreedingEIS). The full system is composed of a web client and a mobile client. The web client is used to name the individual breeding offspring plants and analyze data. The mobile client is based on the technology of widely used smartphones and is suitable for Android and iOS systems. Its functions focus on field evaluation, including quick response code recognition, evaluation data entry, and real-time viewing. In addition, near-field communication technology and portable label machines are introduced to enable breeders to quickly locate each individual plant and accurately label any samples collected from it. Generally, BreedingEIS enables users to accurately and conveniently register phenotypic data and quickly lock target individual plants from large volumes of data. The system provides a low-cost and highly efficient solution for crop information evaluation and enables breeders to better collect, manage, and use breeding data for decision making, which is a valuable resource for crop breeding.

## Introduction

Crop breeding entails improving the genetic characteristics of crops, identifying superior plants, and selecting improved plants using available tools and techniques. Crop breeding is of great importance for the development of crop production, which can not only improve crop yield, enhance crop resistance, and increase beneficial substance content but also improve production efficiency. To establish a new cultivar, the standards of the Distinctness, Uniformity and Stability (DUS) test must be met [1], and whether the DUS criteria are met is usually determined by many generations or years of observation and data recording. Breeders obtain a large amount of data, including data related to phenotype, genotype, and pedigree, during the course of DUS testing. However, crop breeding techniques in most countries are still based on the qualitative experience of breeders. When evaluating phenotypic traits in the field, breeders still use traditional methods, such as manual measurement, empirical evaluation, and written records; however, these methods cause problems, such as great subjectivity, difficulty controlling stability and consistency, a large amount of data traceability and statistical analysis tasks, and low efficiency.

Due to the rapid development and popularization of smartphones and wireless communication technologies, the synchronous transmission of data by equipment dedicated to breeding can be completely based on smartphones and existing 4G/5G wireless communication technologies. Therefore, research on and the application of dedicated crop breeding software has become the main direction for resource research and breeding informatization. Developing specialized breeding equipment and breeding information management software and promoting increased informatization and intellectualization in the breeding process can effectively enhance breeding technology, reduce breeder dependence on qualitative breeding experience, and improve the innovation and utilization of germplasm resources. To date, many studies have reported combining information technology with breeding objectives to improve breeding efficiency [2]. For example, Muehlbauer et al. [3] used computer simulation technology for single seed descent and mass selection methods 40 years ago. Han et al. [4] developed a breeding data collection system based on multilabel recognition technology, greatly improving the efficiency of crop breeding data collection. Jung et al. [5] established a free online breeding information management system (BIMS) to store, manage, archive, and analyze breeding data. Some studies combined genotype and phenotype omics and developed corresponding databases to manage cross platform datasets to help breeding [6–9]. In addition, there are also auxiliary breeding tools that have been developed for single phenotypic indices, such as indices based on imaging [10], root [11], and spectral data [12], or for single species, such as pear [13], rice [14], and wheat [15]. These efforts advance crop breeding to a certain degree. However, there are limitations that come with focusing on a certain breeding link or a complicated operation process without integration and broader adaptability [2]. In addition, breeding often happens outdoors, where the evaluation sample capacity is large. Easily collecting and accurately inputting data in the field is a major problem. Thus far, no studies have reported on whether a breeding system with a wide range of applications can be implemented from the front-end data collection stage to the later data management integration stage by using extensive hardware applicability and a simple and intelligent operation interface.

To address these challenges, in this study, we develop a free, secure new breeding information platform (BreedingEIS, breeding evaluation information system) based on the technology of current popular computers, smartphones, and near-field communication (NFC) tags. We also introduce the basic BreedingEIS principles, design, implementation, and methods of use in detail and point to future development plans for this breeding system.

## System Construction

The system is developed with JavaEE (version 1.8), and browser/server architecture is adopted for centralized deployment and distributed use. The model–view–controller development mode is utilized, and the service-oriented architecture is referred to for functional design. The system is developed using the front- and rear-end separation framework (SpringBoot and Vue), as well as Android and iOS mobile phone operating systems. The back-end service mainly involves 3 programming tools: SpringBoot, Spring, and MyBatis. The front end adopts the Element UI architecture of Vue, and MySQL is employed for data storage to support the principal and subordinate backup of data. In addition, an authority verification mechanism based on the role-based access control model is adopted to encrypt data transmission. Additionally, we introduce NFC technology into the system. We recommend using the NTag216 chip for screening and comparison. This chip was developed by NXP Semiconductors. As a standard NFC label integrated circuit series based on the ISO14443A standard, it has the following advantages: low price, wide applicability, fast communication speed, high security, and flexible card shape and material selection options. Moreover, based on our screening and comparison, we recommend using XP-58IIH (XINYE) as a portable label machine.

## Overview

**Fig. 1.**
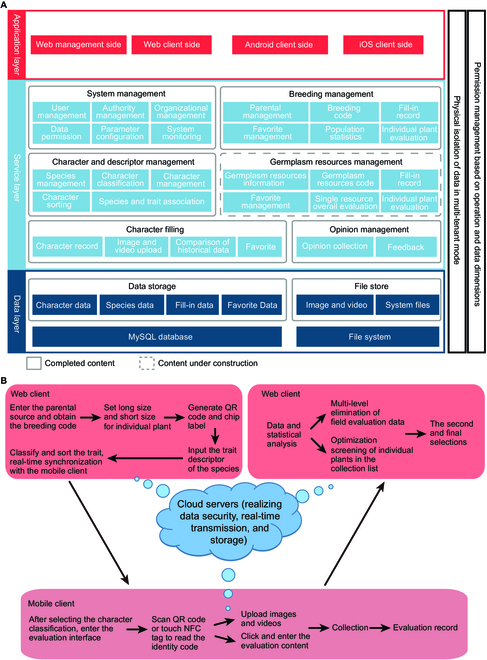
Breeding EIS architecture description (A) and operation flowchart (B).

BreedingEIS has 2 language modes, English or Chinese, and the default setting of the system, which mainly consists of web clients and mobile clients, is in English (Fig. 1). The complete system content is shown in Fig. 1A, and the operation process, which is divided into 3 steps, is shown in Fig. 1B. The operation process is as follows: (a) The user logs into the account, selects the plant species on the web client, enters the parental source information for an individual plant, and obtains the breeding code. The long code (base code + region code + ridge number + line number + serial number) and short code (breeding code + an individual plant tracking number) are set for an individual plant, which together serve as the identity code of that plant to generate a quick response (QR) code and an NFC tag. Then, the trait descriptor of the species is input for evaluation. Next, the plant traits are classified and sorted, and the information is synchronized to the mobile client in real time to prepare for field investigation (Fig. 1B). (b) The user matches the NFC tag generated in the first step with the corresponding individual plant. After selecting the character classification, the user enters the evaluation interface, scans the QR code or touches the NFC tag to read the identity code, uploads images and videos, and clicks and enters the evaluation content. Individual plants with good evaluation indices can be included in the favorite list to facilitate subsequent screening (Fig. 1B). (c) The user enters the web client again for data statistics. Finally, through the multilevel elimination of field evaluation data and the optimization screening of individual plants in the favorite list, the target cultivars are determined by the second and final selections. All data can be exported for plant cultivar patent declaration (Fig. 1B).

## Access Rights of the Account

By setting a 3-level management mechanism (super administrator account, administrator account, and data record user account) for system accounts, we provide various levels of accounts with different permissions to ensure that data can only be shared among relevant users and cannot be accessed by other people (Table). The system sets up a super administrator account with the highest authority, which is mainly used to establish/delete accounts at all levels and carry out system maintenance. One administrator account was set up for each user unit/organization/individual, with the authority to establish/delete the user account of the data record under this account, fill in basic data, generate the barcode, review and count the germplasm resources and hybrid evaluation data of this account, etc. The data record user account is only used to distinguish different filling users, and the authority is mainly to fill in new data and use statistical functions.

**Table.** Authority of each account.

Account type	Establish/delete administrator account	Establish/delete data record user account	Data statistics	Data filling
Super administrator	Yes	Yes	No	No
Administrator	No	Only this account	Only this account	Yes
Data record user	No	No	Only this account	Yes

## Web Client Operating System

**Fig. 2.**
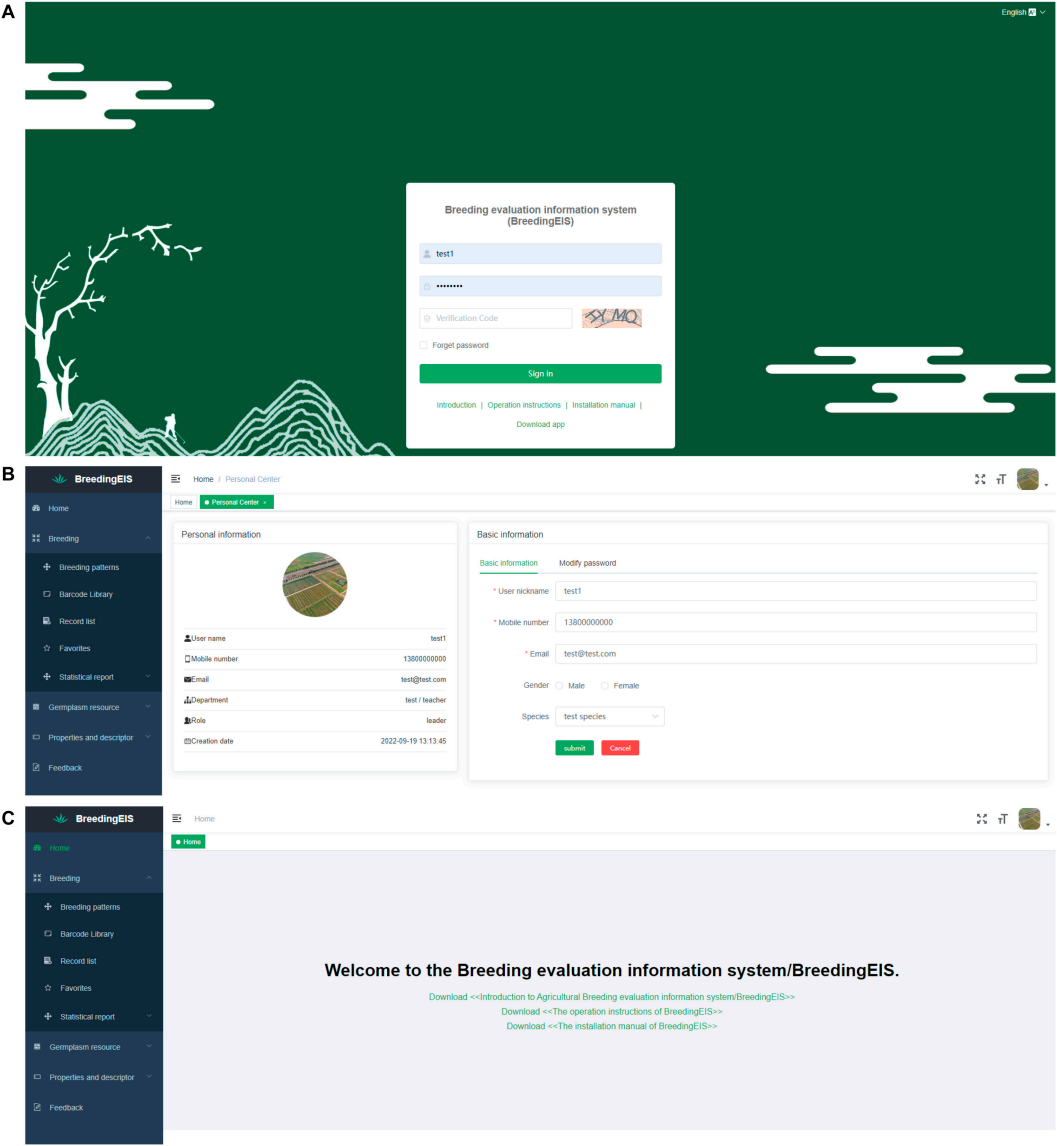
Screenshot of the login interface (A), personal center (B), and homepage (C) on the web client page.

Through the BreedingEIS website (www.nnyshj.com), the user can access the system login page and click the box on the top right to switch between Chinese and English. The user enters the username, password, and verification code and then clicks the sign-in button to enter the system homepage (Fig. 2A). The test account username is test1, and the password is test1234.

**Fig. 3.**
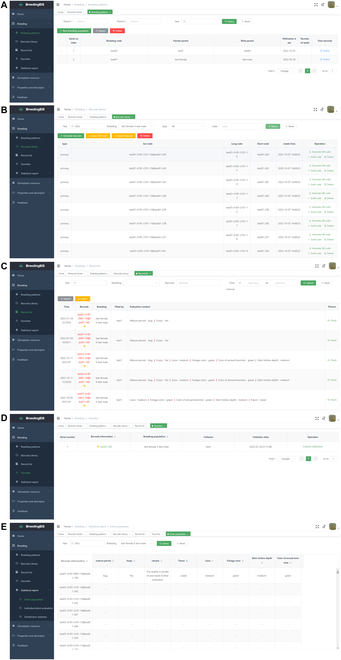
Screenshot of breeding patterns (A), barcode library (B), record list (C), favorites (D), and statistical report (E) subfunctions under the breeding module on the web client page.

**Fig. 4.**
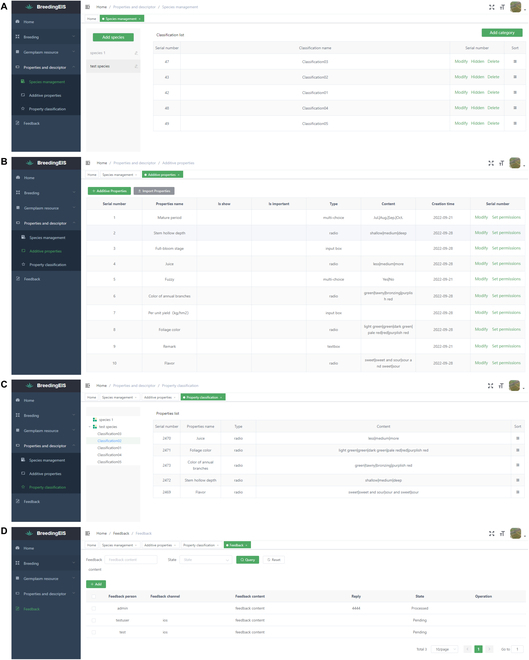
Screenshot of species management (A), additive properties (B), and property classification (C) under the properties and descriptor command and feedback (D) page in the web client page.

After logging in, the user encounters the system homepage, which is divided into a personal center module on the upper right (Fig. 2B), a function selection area on the left, and an operation area on the right (Figs. 2C, 3, and 4). The function selection includes 4 modules: Home (Fig. 2C), Breeding (Fig. 3), Properties and Descriptor (Fig. 4A–C), and Feedback (Fig. 4D). The corresponding functions of each module are as follows:

The personal center module is used to enter the basic information of the account manager and to change the account password (Fig. 2B).

The home module is used to download the introduction file, system deployment protocol, and system application instructions of this system (Fig. 2C).

The breeding module includes 5 subfunctions: breeding patterns, barcode library, record list, favorites, and statistical reports, which are mainly used to comprehensively interpret and apply the information from the breeding patterns, as well as to establish a barcode library and to view the details of existing records (Fig. 3). The breeding patterns are used to manage the existing breeding method, with a focus on the unique numbering for each new group (Fig. 3A). Taking cross breeding as an example, this function can record the details of pollination, seed collection, sowing, planting, and other information relevant to the hybrid combinations. For the users’ convenience, we also added an import function. After downloading the fixed format template, the relevant data are copied to the template according to the template format, which enables the uniform addition of information after uploading. Later, the data for the current year can be filled in according to the phenological periods within a year. The barcode library was used to generate identity codes for individual plants of every population (Fig. 3B). First, the module generates a unique code for an individual plant and records the planting base, region, ridge row, quantity, and other related information. By assigning a unique code to an individual plant and synchronously generating a QR code as one of the certificates for machine recognition, image recognition, or automatic reading by scanning equipment, the system enables the user to automatically process information. Identity codes are the main basis for mobile clients to identify individual plants. To avoid omission, duplication, and redundancy in the work, we adopt a graphical display, which makes it more convenient for users to intuitively understand the coding content and method. Regarding the barcode library, we can easily find all individual plant identity codes and QR codes under a certain breeding method. In addition, the functions of “Export QR code” and “Export barcode information” are added to help users expand their use scenarios. A record list is used to record all breeding data from a detailed analysis of the data in the current user account (Fig. 3C). The system default settings arrange data in reverse order according to the filling time. Users can also quickly locate and view the survey records by filtering according to different criteria, including year, breeding parents, identity code information, and time interval. In addition, the functions of “Export evaluation data” are added to facilitate retention of all evaluation data. The favorite subfunction information is used to mark excellent germplasm resources to facilitate later searches and comparisons and to enable further evaluation screening (Fig. 3D). This subfunction also enables secondary collection. When the user clicks on the favorites button for the first time in the first year/stage, the color changes from white to yellow, and when the user clicks again in the next year/stage, the color changes to red. For potential new cultivars to meet the requirement of stable expression of excellent target traits over the course of 2 years, the next step can be arranged according to the details of the actual situation. This can enable users to track whether an individual plant meets the requirement of a stable expression of excellent target traits over the course of 2 years and can be used as an important candidate for targeting an individual plant. The statistical report subfunction is itself divided into 3 subfunctions: the entire population, individual plant evaluation, and distribution statistics (Fig. 3E). The entire population is used to count all the traits of an individual plant from a single population in a year; that is, to list the detailed evaluation data of all individual plants of the population in a certain year in the form of a table. Individual plant evaluation is used to analyze and compare the evaluation data of individual plants over many years and is often used for in-depth analysis and screening of target traits in population evaluation. Distribution statistics are used to intuitively understand the survey times of an individual plant of a certain population in different years, and they are mainly used to count the fruiting or flowering time and the number of fruits or flowers.

The properties and descriptor module includes 3 subfunctions: species management, additive properties, and property classification (Fig. 4A–C). The species management subfunction mainly involves system compatibility and embeds information about various crop species (Fig. 4A). Users can select the desired species types and add user-defined classifications according to the evaluation schedule at different stages. The subfunction of additive properties is mainly to add and import the characteristics and descriptors required for breeding evaluation of the corresponding crop species (Fig. 4B). This content expands the user’s authority and can be customized according to actual needs to facilitate personalized content evaluation. The property classification subfunction classifies and sorts the characteristics in the above evaluation classification to facilitate the rapid screening of the required characteristics during field evaluation with the mobile client and to improve the efficiency of the system (Fig. 4C).

The feedback subfunction is used to collect users’ suggestions on our system and to answer users’ questions during the use process (Fig. 4D).

## Mobile Client Operating System

**Fig. 5.**
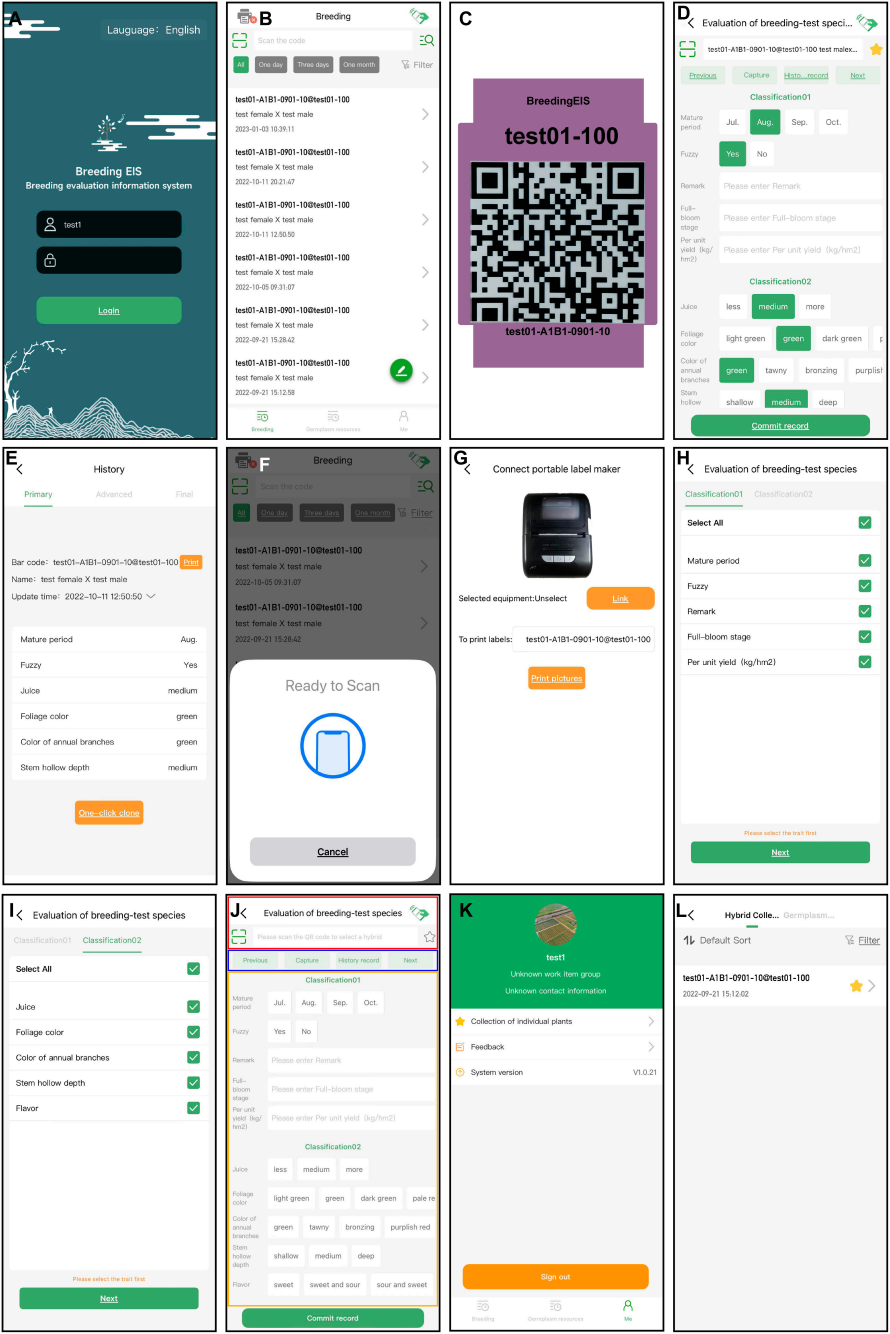
Screenshots from the iOS version of the BreedingEIS system as an example of the mobile client page. (A) Login interface. (B) Homepage. (C) One NFC label with QR code and corresponding evaluation history record information (D, E) as an example. (F) Screen to switch on the NFC function in the iOS system. (G) Screen to switch on the printing function by connecting to the portable label machine through Bluetooth. (H, I) Characteristics grouping selection. (J) Main evaluation page. (K) Personal center. (L) Favorite record.

The user downloads and installs the app package (BreedingEIS-M) from the web client or mobile app store, opens the app, enters the username and password, and clicks Login to enter the main interface (Fig. 5A).

The homepage of the breeding evaluation system is divided into 3 main parts: identity code selection area, screening function area, and record list area (Fig. 5B). In the identity code selection area, the user clicks the scanning function button to scan the QR code label and can quickly locate all the investigation and evaluation records of the corresponding individual plant for the label (Fig. 5B–E). The user can also enter the short code to search by pressing the search function button (Fig. 5B). In addition, the mobile client device can make direct contact, termed “snuggling”, with the NFC tag on this interface to search and locate the evaluation data (Fig. 5B and F). Note that the snuggle function is only applicable to the Android system. The iOS system requires the user to click the “Code scanner” icon on the upper right to call the NFC function (Fig. 5B and F). Moreover, when clicking the printer icon on the top left, the system can connect to the portable label machine through Bluetooth for printing QR codes, which can be attached to the sample for evaluation to facilitate the detection of relevant indicators in the laboratory later (Fig. 5G). In the screening function area, the corresponding investigation and evaluation records can be quickly filtered by one specific day, by a period of 3 days, and by a period of 1 month by clicking the corresponding time button (Fig. 5B). The user can click the filter button to quickly screen the corresponding investigation and evaluation records according to the breeding year, breeding parents, and other information.

The user can click the evaluation function button of the pen icon to enter the breeding evaluation system (Fig. 5B). First, the user selects the characteristic classification for evaluation (Fig. 5H and I). Here, the evaluation classification and the characteristics included in each classification are preset and sequenced through the web. The evaluation classification and the traits contained in each classification are preset and sorted through the web client. The user clicks the upper category button to quickly locate the required category (Fig. 5H and I). The characteristics under each classification can be selected as a group or individually. The main evaluation page is divided into 3 main functional areas for display: (a) The area marked with a red box is the identity code identification function area, as shown in Fig. 5J. The upper end is marked with the corresponding species type, such as “apple”. The lower end is marked with the code scanning function button on the left, the middle part is the identity code display area of the identified individual plant, and the right side is the favorites function button. (b) The area marked with a blue box is the evaluation function area, as shown in Fig. 5J. “Previous” and “Next” correspond to the previous or next identity code in relation to the current identity code. The identity code can be switched directly to simplify the scanning operation process. “Capture” means calling the camera to capture images or record videos. The “History” function can provide a real-time and convenient view of the historical evaluation data and of image or video records for an individual plant corresponding to the current identity code to facilitate comparative evaluation and improve the accuracy and stability of evaluation data. (c) The area marked with a yellow box is a quick evaluation area for characteristics and their descriptors, as shown in Fig. 5J. Different classifications, characteristics, and preset descriptors for this character are displayed in large font. The user directly clicks to complete the record of evaluation content and to integrate characteristics entered in the form of text. After clicking or filling, the user then clicks “Submit Record” at the bottom to upload relevant data, images, and video to the server in real time and to save them in the database in order (Fig. 5J). In addition, by clicking the “Me” button at the bottom of the homepage, the user can enter the personal center (Fig. 5B). Similar to what is seen in the web client, this area provides a feedback function (Fig. 5K). In addition, it also provides access to favorite information and the software version number information (Fig. 5K and L).

## Conclusion and Future Developments

**Fig. 6.**
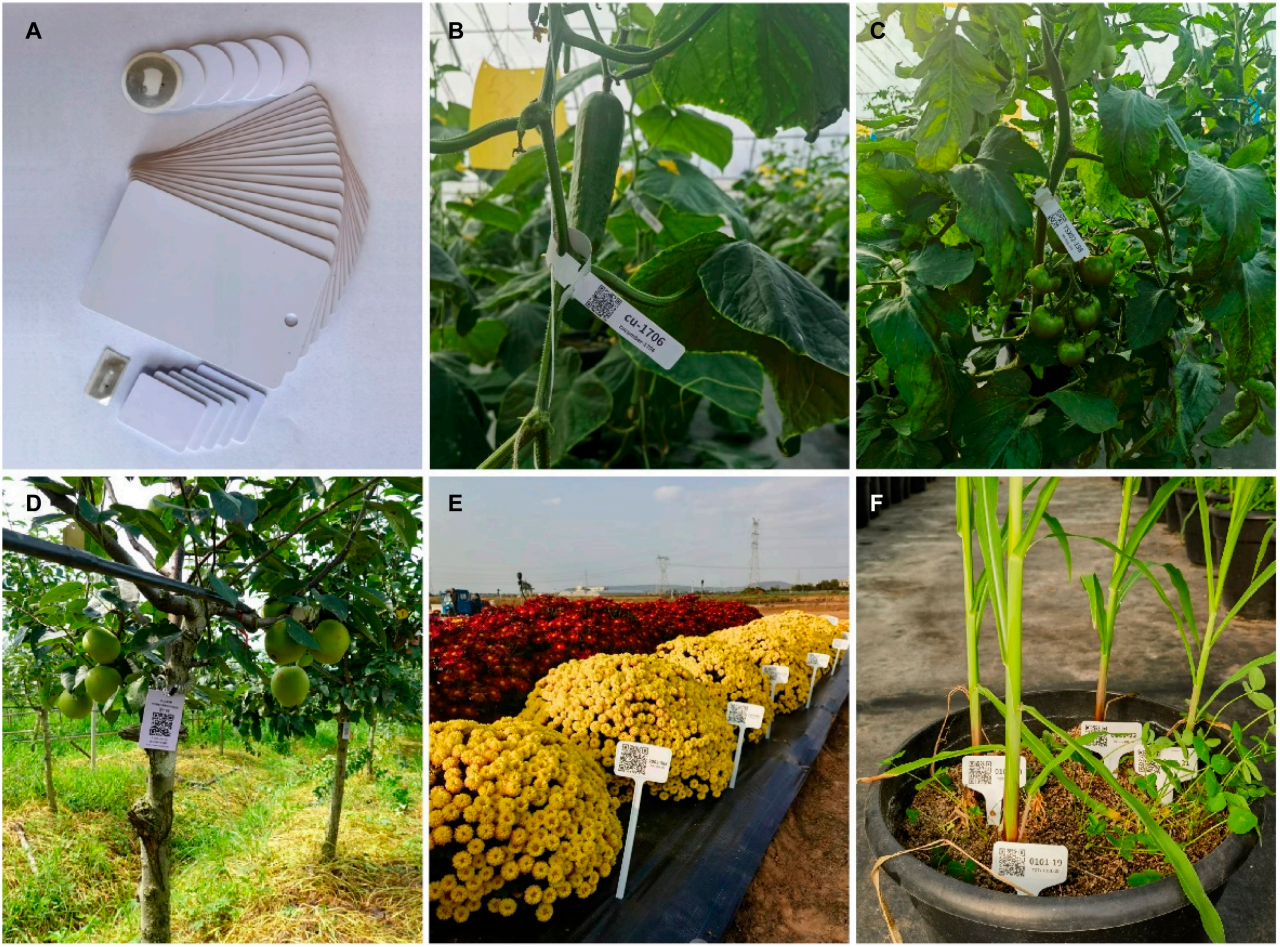
NFC label in different forms (A) and application of BreedingEIS in cucumber (B), tomato (C), pear (D), chrysanthemum (E), and maize (F).

With the rapid development of commercial breeding in recent years, it is especially important to establish a perfect BIMS. In addition to traditional breeding, molecular breeding and identification of important functional genes by genome-wide association studies and quantitative trait locus mapping methods using germplasm resources and genetic populations also rely on a large amount of accurate phenotypic data. Therefore, the informatization of data collection and management is an inevitable trend in breeding work. In this study, we described BreedingEIS, which is a free, safe, easy-to-operate, open-source BIMS. We focus on achieving a system for simple and standardized field data collection, storage, and management and analysis functions, as well as on improving function settings, simplifying operation processes, and improving the application efficiency and popularity of the system to effectively improve breeding efficiency. At present, based on the diversity of Ntag216 chip shapes (Fig. 6A), the system has been successfully applied to breeding research on many crops, such as cucumber (Fig. 6B), tomato (Fig. 6C), pear (Fig. 6D), chrysanthemum (Fig. 6E), and maize (Fig. 6F). However, there are still some limitations, including the addition of more languages, the improvement of germplasm resource information management systems, the integration of laboratory measurement data, and a more complete later-stage data analysis function. We are in the process of establishing the germplasm resource information system. The upload and preliminary analysis functions of the measured data in the laboratory have been applied in the test version. However, we are still exploring the ideal processes for field sampling, indoor measurement, automatic data upload, and integration. In addition, we are actively developing image data analysis and other functions, and we are also attempting to integrate plant phenotypic omics, genomics, environmental science, and other multiomics data to ultimately achieve the goal of highly precise and efficient research and breeding of crops.

## Data Availability

BreedingEIS is publicly available at the web client (www.nnyshj.com) and is built on the open-source BreedingEIS system at Github (https://github.com/qikaijie/BreedingEIS-M). Users can apply to us for company accounts to directly use this system. At the same time, the system also supports full replication to its own server to ensure data privacy. To ensure that users can easily use the system, we also provide a detailed system deployment protocol (the installation manual of BreedingEIS) and system application instructions (the operation instructions of BreedingEIS) on the web client and Github. Meanwhile, we included the application instance of the system on a pear as an example in the operation instructions of BreedingEIS.
